# Carvacrol Encapsulation in Chia Mucilage Nanocapsules Enhances Antimicrobial Activity Against *Escherichia coli* and Preserves Antioxidant Properties in Milk

**DOI:** 10.3390/foods15071196

**Published:** 2026-04-02

**Authors:** Thaís Benincá, Luana Schmidt, Fabíola Ayres Cacciatore, Isadora Altmann Peixoto, Ana Carolina Silveira da Silva, Alina Scherer Pires, Rafaela Diogo Silveira, Juliane Elisa Welke, Patrícia da Silva Malheiros, Paula Rossini Augusti

**Affiliations:** 1 Microbiology and Food Hygiene Laboratory, Food Science and Technology Institute, Federal University of Rio Grande do Sul, Porto Alegre 91501-970, RS, Brazil; thais_beninca@hotmail.com (T.B.); peixotoisa@hotmail.com (I.A.P.); anacarolinasilva2604@gmail.com (A.C.S.d.S.); alinascherer@hotmail.com (A.S.P.); 2 Food Science and Technology Institute, Federal University of Rio Grande do Sul, Porto Alegre 91501-970, RS, Brazil; luana.schmidt020@gmail.com (L.S.); paula.augusti@ufrgs.br (P.R.A.); 3 Microbiology and Food Control Laboratory, Food Science and Technology Institute, Federal University of Rio Grande do Sul, Porto Alegre 91501-970, RS, Brazil; fapoa2017@gmail.com; 4 Toxicology and Food Quality Laboratory, Food Science and Technology Institute, Federal University of Rio Grande do Sul, Porto Alegre 91501-970, RS, Brazil; rafaela_dsilveira@hotmail.com (R.D.S.); juliane.welke@ufrgs.br (J.E.W.)

**Keywords:** carvacrol, nanoencapsulation, antimicrobial activity, antioxidant activity, milk

## Abstract

Carvacrol (CAR) has antimicrobial and antioxidant activity and potential as a food additive, but its intense aroma and high volatility limit its use in foods. Nanoencapsulation has been proposed as a strategy to overcome these limitations. This study evaluated the antioxidant and antimicrobial performance of chia mucilage nanocapsules containing CAR (CMNP) and the effect of nanoencapsulation on CAR content during storage in milk. CMNP and CAR in solution (CS) were added at the Bactericidal Inhibitory Concentration (BIC) and ½ BIC in skim and whole milk. Antioxidant activity was determined by the ABTS (2,2 azinobis (3-ethylbenzothiazoline-6-sulfonic acid) assay, and antimicrobial efficacy was evaluated against *Escherichia coli*. Samples were stored at 37 °C for 48 h to assess antimicrobial activity under optimal growth conditions for *E. coli* and at 5 °C for 14 days to simulate refrigerated storage conditions. CAR was quantified by HS-SPME–GC/MS. CMNP increased antioxidant activity compared to the control, showing values similar to CS. At 37 °C, CMNP inhibited *E. coli* to undetectable levels using ½ BIC in both milks. At 5 °C, the full BIC was required in whole milk, while ½ BIC was sufficient in skim milk. Nanoencapsulation resulted in lower initial CAR content but promoted gradual release during storage at 5 °C and 37 °C. CMNPs show potential to contribute to the microbiological safety and oxidative stability of milk.

## 1. Introduction

Global demand for milk and dairy products has been steadily increasing, reinforcing the importance of ensuring their microbiological safety and oxidative stability. Despite this, the consumption of raw milk and unpasteurized dairy products remains common worldwide. Moreover, fresh cheeses, due to their physicochemical characteristics such as high moisture content, provide favorable conditions for the survival and growth of foodborne pathogens, such as *Escherichia coli* [1,2]. Another significant challenge for the quality and safety of dairy products is the occurrence of oxidative processes, especially lipid oxidation, which can compromise sensory characteristics, reduce nutritional value, generate undesirable compounds during storage, and reduce shelf life [3].

*E. coli* is the most prevalent facultative aerobic bacterium in the intestinal microbiota of warm-blooded animals and is among the primary contaminants of milk and dairy products [4]. Although most *E. coli* strains are not pathogenic, some serotypes, such as *E. coli* O157:H7, can cause serious illness in humans due to the production of Shiga toxin (STEC) [5]. Furthermore, *E. coli* is an important indicator of hygiene failures during food handling, as its presence suggests possible fecal contamination, reflecting inadequate practices by handlers [4]. It is also widely used as a model organism in microbiological studies and is recognized as a representative of Gram-negative bacteria [6]. Therefore, identifying natural antimicrobial agents capable of reducing *E. coli* proliferation in dairy systems is of considerable importance.

Carvacrol [(2-methyl-5-(1-methyl) phenol)—CAR] is a monoterpenoid extracted mainly from the essential oils of oregano and thyme [7]. Due to its aroma, it is approved as a flavoring agent in China, the United States, and the European Union [8,9] and has been described as safe for humans at a daily oral intake of up to 2 mg kg^−1^, for 30 consecutive days [10].

CAR exhibits antimicrobial action against important foodborne pathogens such as *Salmonella*, *Listeria monocytogenes*, and *E. coli* [11,12]. Additionally, it demonstrates antioxidant potential due to its capacity to scavenge reactive oxygen species (ROS), preventing oxidative degradation of food proteins and lipids [13]. However, the practical application of CAR in dairy products remains limited because of its strong and persistent odor and high volatility, which can lead to sensory alteration and loss of both antimicrobial and antioxidant efficacy.

To overcome these drawbacks, nanoencapsulation strategies have been explored to mitigate the compound’s aroma, protect it from volatilization, and improve its stability and dispersion within food matrices [12,14,15,16]. Recent studies have also shown that nanoemulsion-based systems can enhance the stability and antimicrobial performance of CAR in milk. For instance, Li et al. (2025) [17] demonstrated that sodium caseinate/hydroxipropyl-β-cyclodextrim nanoemulsions improved the antibacterial efficacy of CAR against *Bacillus cereus* and maintained good physical stability of milk during storage. These results highlight the growing interest in delivery systems capable of improving CAR functionality in dairy matrices.

In this context, evaluating milk with different fat contents, such as whole and skimmed milk, is relevant to better understand how fat may influence the availability and overall performance of hydrophobic compounds such as CAR.

Various encapsulation systems, such as nanoliposomes, nanoemulsions and Eudragit-based nanocapsules [11], have been investigated. In particular, chia mucilage has attracted particular attention because of its natural origin, biocompatibility and ability to reduce the aroma of CAR while enhancing its antimicrobial efficacy in lettuce, cabbage and grapes [12,14,15,16]. Despite these promising results, no study to date has evaluated the antimicrobial and antioxidant effects of CAR nanoencapsulated in chia mucilage in milk. Furthermore, the temporal retention and released behavior of CAR from chia mucilage nanocapsules (CMNP) has not yet been investigated in dairy systems.

Therefore, this study aimed to evaluate the antimicrobial activity of CMNP against *E. coli* and its antioxidant potential in milk, as well as investigate the time-dependent retention and release behavior of CAR in this matrix. Furthermore, it compares the performance of CMNP in matrices with different fat contents, offering new insights into how food composition affects the functionality of encapsulated bioactive compounds. To the best of our knowledge, this is the first study to investigate these combined aspects in a dairy matrix. By linking release kinetics with microbiological outcomes under realistic storage conditions, this study provides relevant insights for the application of nanoencapsulated CAR in dairy systems.

## 2. Materials and Methods

### 2.1. Materials

Chia seeds, whole milk powder, and skimmed milk powder were obtained from a local market (Porto Alegre, Brazil). CAR (CAS: 499-75-2) (≥98%) was purchased from Sigma-Aldrich (St. Louis, MO, USA). A bacterial cocktail containing three strains of *E. coli* (*E. coli* ATCC 8739, *E. coli* ATCC 25.922 and *E. coli* DH5–α) was obtained from the bacterial culture collection of the Food Microbiology and Control Laboratory (ICTA/UFRGS, Porto Alegre, Brazil). Polysorbate-80 (≥99%) and acetic acid (99.8%) were purchased from Neon (Suzano, Brazil), and absolute ethanol (99.5% PA) from Dinâmica (São Paulo, Brazil). Peptone water and Brain Heart Infusion (BHI) broth was obtained from Kasvi (São José do Pinhais, Brazil), and Violet Red Bile Agar (VRBA) was obtained from Himedia (Thane, Maharashtra, India). ABTS radical (2,2 azinobis (3-ethylbenzothiazoline-6-sulfonic acid) was purchased from Sigma-Aldrich (São Paulo, Brazil) and potassium persulfate was obtained from Scientific Exodus (São Paulo, Brazil). Analytical standard CAR (code 42632, 98% purity) was obtained from Sigma-Aldrich (Steinheim, Germany). NaCl was obtained from Nuclear (São Paulo, Brazil), and helium (analytical purity, 99.999%) from Linde Gases (Canoas, RS, Brazil).

### 2.2. Preparation of CAR Solution and CMNP

An emulsion of CAR in water, referred to as CAR in solution (CS), was prepared by mixing 1.06 g of CAR and 0.1 g of polysorbate-80 in 10 mL of Milli-Q water. The mixture was then stirred and stored under refrigeration (5 °C) until use.

Chia mucilage was prepared according to Dick et al. (2015) [18] with modifications. The chia seeds were immersed in distilled water at a ratio of 1:30 (*w*/*v*) and then shaken using a suspended mechanical shaker (EDUTEC EEQ-9034, Curitiba, Brazil) for 2 h at room temperature (25 °C). The mucilage suspension formed was separated from the seeds by centrifugation at 9000× *g* for 30 min (HITACHI—High Speed Refrigerated Centrifuge CR 21G III, Tokyo, Japan) and then filtered using a vacuum pump (Model 355. B. 1, Quimis, LTDA, Diadema, SP, Brazil) and a sieve (1 mm) to remove the seeds. The resulting mucilage gel was dried in an oven at 60 °C (De Leo TLK48, São Paulo, Brazil) with air circulation for 18 h and then stored at room temperature (25 °C) in sealed plastic bags until use.

CAR was encapsulated in chia mucilage according to the methodology described previously [12]. Briefly, dried chia mucilage was rehydrated in distilled water using a magnetic stirrer (WARMNEST 78HW-1, Araucária, PR, Brazil) for 2 h at 25 °C, acidified with acetic acid (1 M) to pH 4.0 and autoclaved (aqueous phase). At the same time, an organic phase was prepared containing CAR (40 mg), polysorbate-80 (13.5 mg) and ethanol (4 mL). The organic phase was then added drop-by-drop to the aqueous phase during homogenization (8000 rpm/15 min) in an Ultra Turrax^®^ (digital model T25; IKA, Staufen, Germany). This process produced 24 mL of solution (concentration 1.67 mg mL^−1^). Furthermore, blank chia mucilage nanocapsules (UCMN) were prepared following the same procedure as CMNP, replacing CAR with caprylic acid, a compound known to lack antimicrobial activity, to serve as an appropriate control.

### 2.3. Characterization of CMNPs

The CMNPs were characterized in terms of mean diameter, polydispersity index (PDI), and zeta potential. The mean diameter and PDI were determined using dynamic light scattering. The zeta potential was determined using the electrophoretic mobility, both using the Zetasizer^®^ equipment (Nano Series, model ZEN 3600, Malvern Instruments, Egham, UK), after diluting the samples in ultrapure water at a ratio of 1:5.

### 2.4. Determination of the Bactericidal Inhibitory Concentration (BIC) of CS and CMNP Against E. coli

Bacteria strains were grown separately in BHI broth at 37 °C for 18 to 24 h under aerobic conditions. For the tests, each suspension was adjusted approximately 8 log CFU mL^−1^ by dilution in BHI broth to obtain an optical density of 0.5 (OD 630 nm) using a UV spectrophotometer (BEL Engineering^®^, model M51, Monza, Italy). Subsequent decimal dilutions were made in BHI broth until the desired concentration was reached (5–6 log CFU mL^−1^). To prepare the bacterial cocktail, 3 mL of each *E. coli* strain was combined in a sterile tube, resulting in a final volume of 9 mL.

The BIC of CS and CMNP was determined for the individual *E. coli* strains and for the cocktail composed of the three strains, according to Cacciatore et al. (2022) [12]. In this study, BIC was used as an operational parameter to estimate the antimicrobial concentration required to achieve complete bacterial inactivation under the experimental conditions. While the Minimum Bactericidal Concentration (MBC) is commonly defined as the concentration capable of reducing the bacterial population by at least 3 log CFU, the BIC corresponds to the lowest concentration of the antimicrobial able to eliminate detectable viable cells after incubation and plating.

Briefly, samples of CS and CMNP were evaluated in 96-well microplates. A positive control (without antimicrobials) and a negative control (without inoculum) were included. The plates were incubated at 37 °C for 24 h. Subsequently, 20 µL from each well was plated on VRBA and incubated at 37 °C for 24 h. Growth of typical colonies was observed, and the lowest concentration showing complete inactivation of the target bacteria was recorded as the BIC.

### 2.5. Application of CMNP and CS on Milk Samples

Milk samples were prepared in a standardized manner for all analyses described in this study. Formulations containing CMNP and CS, in concentrations previously determined as BIC (Table S1), were added to sterile tubes containing skimmed and whole milk powder, reconstituted to a total of 10 mL per tube. Experimental groups consisted of CS; CMNP; CS + skimmed milk; CMNP + skimmed milk; skimmed milk only; CS + whole milk; CMNP + whole milk; and whole milk only. This same preparation was used in all subsequent experimental steps immediately after homogenization.

### 2.6. Evaluation of In Vitro Antioxidant Activity of Milk Samples Containing CMNP and CS

The ABTS radical scavenging activity was determined according to the methodology described by Rufino et al. (2007) [19]. Briefly, the ABTS radical was prepared by reacting 5 mL of the ABTS stock solution with 88 µL of potassium persulfate. The mixture was kept in the dark at room temperature for 16 h. Afterwards, 1 mL of this mixture was diluted in absolute alcohol. In a dark environment, 30 µL of sample from groups described above was transferred to test tubes containing the ABTS radical and homogenized. The absorbance of the samples was read at 734 nm on a BEL Engineering^®^ UV spectrophotometer (model M51, Monza, Italy) after 6 min of reaction. The antioxidant activity of the samples was expressed as the percentage of ABTS radical inhibition.

### 2.7. Efficacy of CMNP and CS in Reducing E. coli in Milk

Milk samples containing CMNP and CS, as previously described, were contaminated with an *E. coli* cocktail to achieve an initial count of approximately 4 logs CFU mL^−1^. Positive (milk with bacterial inoculum) and negative (milk without inoculum) controls were included. The tubes were incubated at 5 °C for 0, 2, 5, 10, and 14 days, and at 37 °C for 0, 2, 4, 6, 12, 24, and 48 h. Quantification of *E. coli* was performed using the spread-plate method on VRBA, followed by incubation at 37 °C for 24 h [20].

### 2.8. Time-Dependent Quantification of CAR in Milk Treated with CS and CMNP

The concentration of CAR was evaluated in whole and skim milk to verify the potential of encapsulation in retaining the compound. Samples were packaged in 15 mL Falcon^®^ tubes, kept hermetically sealed throughout the experimental period, and stored at 37 °C (evaluation points at 0, 2, 6, 12, 24, and 48 h) and at 5 °C (evaluation points at 0, 2, 5, 10, and 14 days). For the analyses, 1 mL aliquots were sequentially withdrawn from the same tube at each evaluation time, ensuring that the monitoring of CAR concentration was carried out over time from a single experimental unit per condition.

CAR was extracted by headspace solid-phase microextraction (HS-SPME) with a 2 cm divinylbenzene/carboxene/polydimethylsiloxane (DVB/Car/PDMS, 50–30 µm, Supelco, Bellefonte, PA, USA) fiber, following a previously optimized protocol: 1 mL of sample and 0.3 g of NaCl (*w*/*v*, Nuclear, São Paulo, Brazil), at 55 °C for 45 min [21]. The CAR content in milk was quantified by gas chromatography–mass spectrometry with a quadrupole GC/qMS analyzer (-QP2010, Shimadzu, Kyoto, Japan) as described by Tópor et al. (2025) [16]. Briefly, the compounds were separated using a polyethylene glycol column (DB-Wax, 30 m × 0.25 mm × 0.25 µm; J&W Scientific INC., Folsom, CA, USA). The GC oven was maintained at 35 °C for 5 min, followed by heating to 140 °C at a rate of 3 °C min^−1^, reaching a final temperature of 240 °C at 20 °C min^−1^. The injector and detector temperatures were maintained at 240 °C, the helium flow rate was 1 mL min^−1^, and the desorption was performed in splitless mode. The analytical determination was performed in triplicate.

A curve containing 7 points (y = 0.014x + 1.3047, R^2^: 0.9985), at concentrations ranging from 0.5 to 500 µg L^−1^, was obtained in a milk model solution using the CAR analytical standard. Validation of the HS-SPME-GC/qMS method followed the criteria established by the International Conference on Harmonization [22].

### 2.9. Statistical Analysis

For the determination of BIC, bacterial count in milk, and CAR quantification, the analyses were conducted in three independent experiments in three different weeks. For the analyses of antioxidant activity, two independent experiments were conducted in two consecutive weeks. The analyses were performed using GraphPad Prism software version 5.0 (GraphPad Software, Inc., La Jolla, CA, USA). The obtained data were subjected to Analysis of Variance (ANOVA), and Tukey’s test was applied when necessary. For all data, differences were considered significant at *p* < 0.05.

## 3. Results and Discussion

### 3.1. CMNP Characterization

The mean diameter of CMNP was 186.0 ± 26.7 nm, which is within in the range typically defined for nanoparticles (up to 1000 nm), as described by Jeevanandam et al. (2018) [23]. In this work, the PDI of the nanocapsules was 0.302 ± 0.03, and the zeta potential showed a negative value of −9.655 ± 0.48 mV. PDI indicates the degree of uniformity in particle size, and values below 0.3 are generally associated with monodisperse systems, while slightly higher values suggest a broader size distribution. Therefore, although the value obtained in this study is slightly above 0.3, it still indicates a relatively homogeneous distribution, with no evidence of pronounced polydispersity. Zeta potential represents the electrical charge on the surface of nanoparticles and influences their colloidal stability [24]. However, it is important to note that zeta potential alone does not definitively predict nanoparticle stability, as other factors such as steric effects and medium composition can also influence colloidal behavior [25].

### 3.2. Effect of CMNP and CS on Antioxidant Capacity in Milk

**Figure 1:**
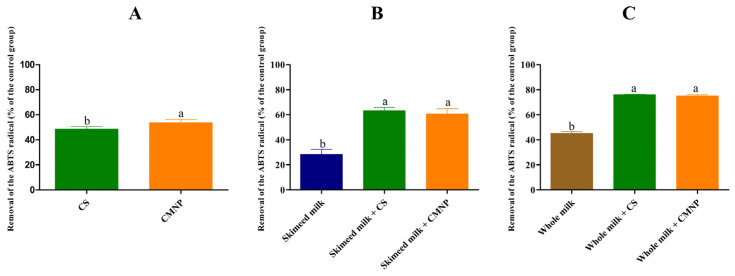
Percentage of ABTS radical removal from CS (1.33 mg mL^−1^) and CMNP (0.83 mg mL^−1^) (**A**), skimmed (**B**) and whole milk (**C**) treated with CS (1.33 mg mL^−1^) and CMNP (0.83 mg mL^−1^). CMNP—Chia mucilage nanocapsules with carvacrol, CS—Carvacrol in solution. Different letters indicate significant differences between treatments (*p* < 0.05). Data represent the mean ± standard deviation of analyses performed in duplicate.

In the present study, antioxidant activity was evaluated through the ABTS assay. This method determines how effectively a substance can reduce the artificially generated ABTS radical cation (ABTS^++^) [26]. In milk-free assays, CMNP showed greater antioxidant activity compared to CS (53.71 vs. 48.66%, Figure 1A; *p* < 0.05). This enhancement in antioxidant capacity due to nanoencapsulation has been previously reported for other delivery systems. For example, gold and silver nanoparticles loaded with Mentha Spicata essential oil exhibited higher antioxidant activity in scavenging the ABTS radical compared to the free essential oil [27]. Hadidi et al. (2020) [28] also reported higher antioxidant activity for chitosan nanoparticles loaded with clove essential oil (15.9–71.8%) compared to the free oil (15.4–60.4%). This increase in antioxidant activity can be explained by the protective effect of encapsulation, which protects the bioactive compound against adverse effects such as oxygen and temperature, decreasing the evaporation rate and releasing it in a controlled manner during the assay [28,29].

In milk-containing assays, both CS and CMNP were able to remove a significantly higher percentage of the ABTS radical compared to untreated milk samples (*p* < 0.05; Figure 1B,C). However, no statistical difference was observed in this antioxidant activity between CS and CMNP when added to whole milk and skimmed milk. These findings suggest that both treatments enhanced the antioxidant potential of milk, consistent with the well-established antioxidant efficacy of CAR. A similar result was reported by Ben Jemaa et al. (2017) [30], who observed that both free thyme essential oil and its nanoemulsion protected semi-skimmed milk against oxidation through the DPPH radical method.

The similar antioxidant effect of the use of CS and CMNP in whole and skimmed milk may be associated with the interaction of phenolic compounds (such as CAR) with milk proteins, which would result in phenolic compound trapping and increased stability. This increase stability most likely occurs due the reduced availability of phenolic moiety for oxidative degradative reactions [31]. Moreover, this interaction with milk proteins was already described for phenolic compounds from Turkish Tombul hazelnut and resulted in improvement in antioxidant ability of the phenolic extracts [32]. Thus, both free and nanoencapsulated CAR in chia mucilage appear to be promising strategies to enhance the oxidative stability of dairy products, contributing to quality preservation and shelf-life extension.

### 3.3. Efficacy of CMNP and CS in Reducing E. coli in Milk

The BIC of CMNP against individual strains and against the *E. coli* cocktail was 0.83 mg mL^−1^, which was lower than CS (1.33 mg mL^−1^) (Table S1). This result confirms that nanoencapsulation potentiated the antimicrobial action of CAR, reducing the concentration of the active compound required to inactivate the target bacteria, as previously described [12].

**Figure 2:**
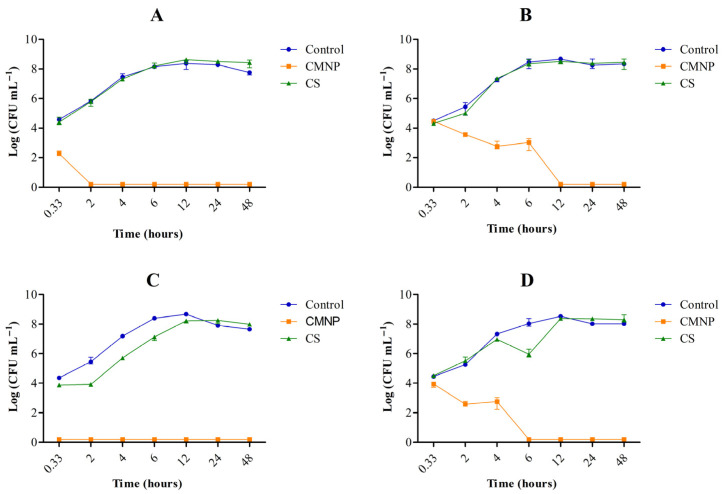
Survival of *Escherichia coli* in whole milk tested with (**A**) BIC and (**B**) ½ BIC and skimmed milk tested with (**C**) BIC and (**D**) ½ BIC at 37 °C. CMNP—Chia mucilage nanocapsules with carvacrol, CS—Carvacrol in solution. Data represent the mean ± standard deviation of analyses performed in triplicate.

In this work, CMNP and CS were added to skimmed and whole milk containing the *E. coli* cocktail, at concentrations corresponding to their BIC (1.33 and 0.83 mg mL^−1^) and ½ BIC (0.66 and 0.42 mg mL^−1^). The antibacterial activity of CS and CMNP against *E. coli* in skimmed and whole milk was initially evaluated at 37 °C (Figure 2), since this is the ideal temperature for the multiplication of the bacteria.

The viable counts of *E. coli* in whole milk treated with BIC and ½ BIC of CMNP decreased to levels below the detection limit in 2 and 12 h, respectively (Figure 2A,B). In skimmed milk, the reduction in viable *E. coli* counts treated with BIC of CMNP was immediate (Figure 2C) and with ½ BIC of CMNP they decreased to levels below the detection limit in 6 h (Figure 2D). However, CS did not show a similar antibacterial effect in milk samples. The number of viable bacteria in the CS group increased from 4 logs CFU mL^−1^ to 8 logs CFU mL^−1^ (*p* > 0.05) after 48 h of incubation, in both whole and skimmed milk. Similar results were reported by Li et al. (2025) [17], who demonstrated that CAR nanoemulsions added to whole and skimmed milk decreased the viable counts of *Bacillus cereus* by 6 logs CFU mL^−1^, with no viable bacteria detected after 24 h at 37 °C. In contrast, free CAR led to an increase in *B. cereus* counts from 6 to 7 logs CFU mL^−1^ after 24 h at 37 °C in both milk types.

**Figure 3:**
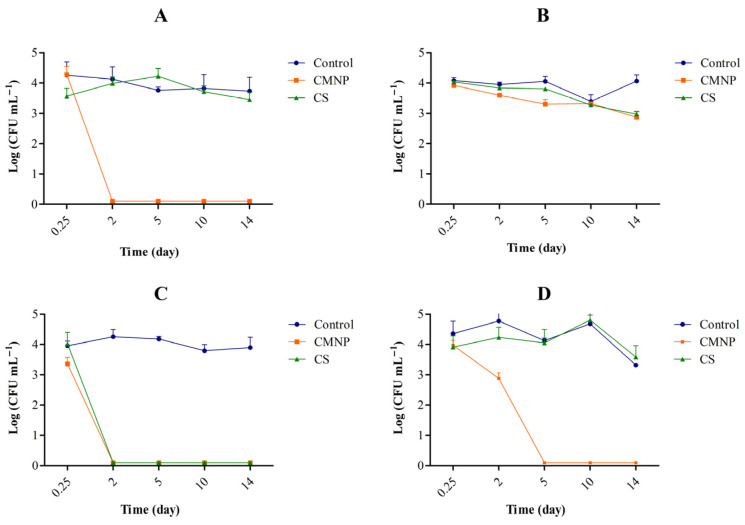
Survival of *Escherichia coli* in whole milk tested with (**A**) BIC and (**B**) ½ BIC and skimmed milk tested with (**C**) BIC and (**D**) ½ BIC at 5 °C. CMNP—Chia mucilage nanocapsules with carvacrol, CS—Carvacrol in solution. Data represent the mean ± standard deviation of analyses performed in triplicate.

The effect of CAR (in free and nanoencapsulated form) on the behavior of *E. coli* in skimmed and whole milk under refrigeration (5 °C) was also investigated (Figure 3). In whole milk, only CMNP treatment at BICs reduced *E. coli* counts to below the method detection limit from the second day of storage (Figure 3A). The results showed that it is necessary to use CMNP at the BIC concentration in whole milk to reduce the *E. coli* population (4.5 logs CFU mL^−1^) at 5 °C. On the other hand, in skimmed milk, the application of ½ BIC was sufficient to keep the *E. coli* population below the detection limit of the method (Figure 3D). These findings demonstrate that CMNP presented significant bactericidal effects in whole milk, indicating that nanocapsules containing CAR are promising for application in the manufacture of cheeses, which are usually produced with this type of milk. Similar behavior was reported for thymol nanoemulsion produced with a gelatin–lecithin mixture, which reduced *E. coli* O157:H7 to below the detection limit after 48 h in whole milk kept at 21 °C [33].

Without addition of CAR (control), *E. coli* counts remained between 4 and 4.5 logs CFU mL^−1^ for up to 14 days for both types of milk. In skimmed milk, treatments with CS and CMNP at BICs reduced *E. coli* counts below the detection limit of the method from the second day of storage (Figure 3C). Furthermore, no significant differences were observed between CMNP and CS (BIC) treatments in skimmed milk (*p* < 0.05), indicating that both systems were similarly effective in controlling bacterial growth under these conditions. This behavior suggests that, in skimmed milk under refrigeration, the initial availability of free CAR may have been sufficient to inactivate the *E. coli* population before a substantial decrease in measurable levels occurred, as supported by the time-dependent reduction in measurable CAR levels. In contrast, in whole milk interactions, the lipid fraction may have reduced the availability of free CAR, limiting its antimicrobial efficacy under the same conditions [34]. These interpretations are proposed with caution, as these mechanisms were not directly evaluated in the present study.

At ½ BICs (Figure 3D), the addition of CMNP (0.42 mg mL^−1^) to skimmed milk reduced 2 logs CFU mL^−1^ of *E. coli* on day 2 compared to the control (*p* < 0.05) and 1 log CFU mL^−1^ compared to CS (0.66 mg mL^−1^). From day 5 of storage onwards, CS maintained counts similar to those of the control, while CMNP (½ BIC) alone reduced the *E. coli* population to undetectable levels.

The main difference between skimmed and whole milk is the amount of fat present. Whole milk must contain at least 3% fat, while skimmed milk can have a maximum of 0.5% of this component [35]. The amount of fat in milk modifies not only the flavor and texture, but also the interaction of bioactive compounds, such as CAR, with the food matrix. Hydrophobic compounds such as essential oils and their constituents, including CAR, have an affinity for lipids (such as fat globules from milk), which reduces their availability due to the binding to these components [36]. Thus, CAR antimicrobial efficacy is reduced in high-fat foods, such as whole milk, when compared to their action in culture media [36,37,38]. In addition, the fat present in milk can protect microorganisms, forming a protective layer that prevents CAR from contacting the bacterial cell [37].

Nanoencapsulation of CAR can improve its availability and stability, allowing for a more gradual and sustained diffusion of the compound through the milk matrix. Moreover, nanoscale CAR droplets facilitate the penetration into the cell wall and outer membrane of bacteria, reducing the energy required for their disruption and resulting in cell death [17,39].

### 3.4. Time-Dependent Quantification and Retention of CAR in Milk Treated with CMNP and CS

A time-dependent quantification of CAR by HS-SPME–GC/MS was carried out to estimate CAR retention and its allowed estimation of its apparent release behavior in milk over time. The HS-SPME–GC/MS method showed repeatability and intermediate precision with relative standard deviations below 6% and 8%, respectively, and recoveries above 92%. The limit of detection (LOD) (0.25 µg L^−1^) and limit of quantification (LOQ) (0.5 µg L^−1^) confirmed adequate sensitivity according to ICH (2005) [22].

**Figure 4:**
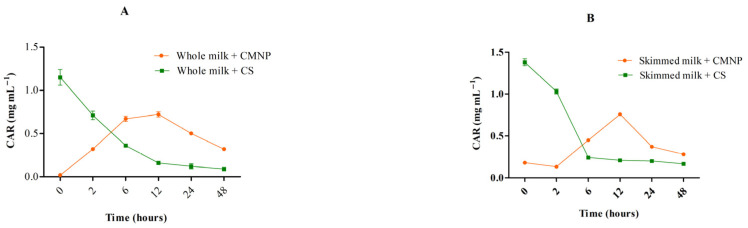
Time independent quantification of CAR in milk treated with (**A**) CMNP and CS in whole milk and (**B**) CMNP and CS in skimmed milk at 37 °C. CMNP—Chia mucilage nanocapsules with carvacrol, CS—Carvacrol in solution. Data represent the mean ± standard deviation of analyses performed in triplicate.

CAR levels were determined in whole and skimmed milk treated with CS and CMNP at BICs and incubated at 37 °C for 48 h (Figure 4A,B). Milk samples treated with CS presented the highest apparent dispersion immediately after addition 1.15 mg mL^−1^ in whole and 1.38 mg mL^−1^ in skimmed milk (*p* < 0.05). The lowest release level in whole milk was at 12 h (*p* < 0.05) and in skimmed milk was at 6 h (*p* < 0.05), followed by a marked reduction over 48 h, reaching 0.09 and 0.17 mg mL^−1^ (*p* < 0.05), respectively. These results suggest that CS suffered rapid volatilization which likely reduced its availability in milk samples. On the other hand, milk samples added with CMNP showed a gradual increase in measurable CAR over time, reaching the highest detected concentrations at 6 h for whole (0.70 mg mL^−1^) and 12 h skimmed milk (0.76 mg mL^−1^).

**Figure 5:**
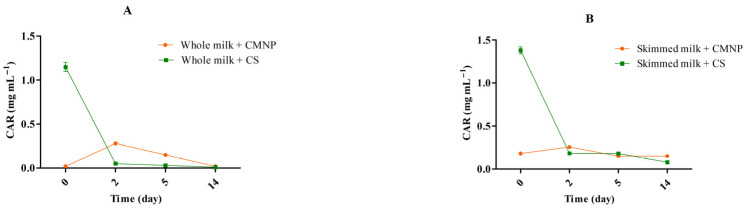
Time independent quantification of CAR in milk treated with (**A**) CMNP and CS in whole milk and (**B**) CMNP and CS in skimmed milk at 5 °C. CMNP—Chia mucilage nanocapsules with carvacrol, CS—Carvacrol in solution. Data represent the mean ± standard deviation of analyses performed in triplicate.

At 5 °C (Figure 5A,B), the same pattern was observed. In skimmed milk, CAR from CMNP was gradually detected throughout refrigerated storage (0.18 to 0.15 mg mL^−1^), with the highest value (0.25 mg mL^−1^, *p* < 0.05) on day 2. In whole milk, CMNP resulted in lower detectable CAR at time zero and day 14 (0.02 mg mL^−1^, *p* < 0.05) and a peak on day 2 (0.28 mg mL^−1^, *p* < 0.05). This difference suggests that fat interferes with CAR availability, likely due to its affinity for the lipid fraction of whole milk (3% fat), while skimmed milk (0.5% fat) exhibited less binding. For CS, a sharp decline in detectable CAR was also observed over time, dropping from 1.38 and 1.15 mg mL^−1^ at time zero to 0.08 and 0.01 mg mL^−1^ for skimmed and whole milk, respectively, after 14 days. This pronounced reduction in both systems indicates that factors other than fat content play a major role in CAR loss during refrigerated storage. This behavior can be primarily attributed to the intrinsic physicochemical properties of CAR, particularly its high volatility [40], and its ability to diffuse through the matrix and be released to the surrounding atmosphere, even under refrigerated conditions, as demonstrated in protein-based systems [41]. In non-encapsulated systems, CAR can freely diffuse within the liquid matrix and migrate toward the headspace, contributing to its progressive loss during storage. This behavior is consistent with the known vapor-phase activity of CAR and other essential oil components in food systems [42]. In addition, CAR may partition between the aqueous phase and lipid components of the matrix, which can influence its distribution and availability [34,43]. Although fat may influence CAR distribution, the consistent decline observed in both skimmed and whole milk suggests that volatilization and diffusion are the dominant mechanisms.

These temporal trends align with the microbiological data at 5 °C (Figure 3A,C), where CMNP maintained *E. coli* counts below the detection limit from day 2 onward, precisely the period corresponding to the highest measurable CAR levels. All these differences observed between CS and CMNP in milk samples possibly occurred due the protective effect of nanoencapsulation, which preserved CAR during storage and maintained its antimicrobial efficacy in milk.

**Figure 6:**
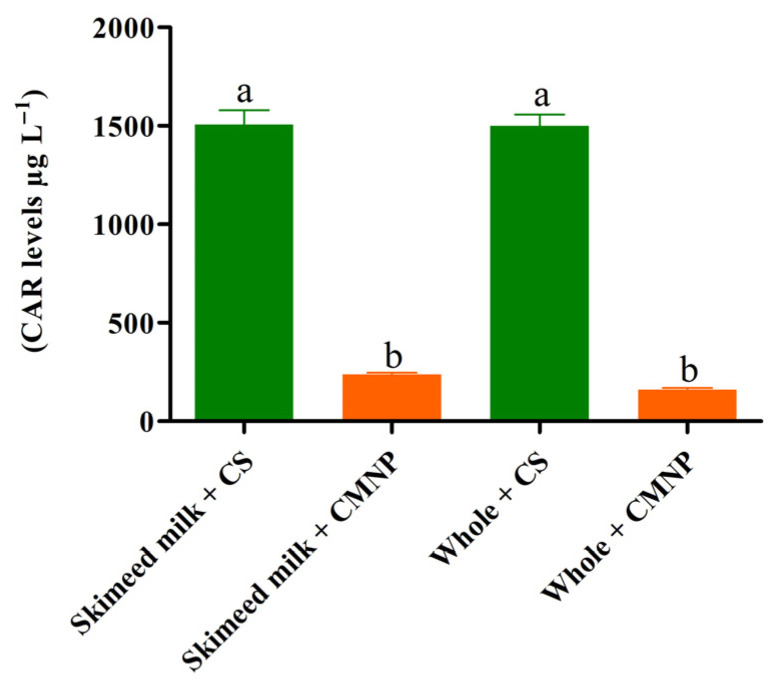
CAR levels in milk treated with CS and CMNP at CIB concentrations. CMNP—Chia mucilage nanocapsules with carvacrol. CS—Carvacrol in solution. Different letters indicate significant differences between treatments (*p* < 0.05). Carvacrol levels correspond to the mean ± standard deviation of HS-SPME-GC/qMS analyses performed in triplicate.

Figure 6 presents the final CAR concentrations in milk treated with CS and CMNP. In CS treated samples, CAR reached 1506 ± 127 µg L^−1^ (skimmed) and 1498 ± 102 µg L^−1^ (whole milk), approximately 37 times higher than the odor threshold (40 µg L^−1^) reported by Tópor et al. (2025) [16] for grapes, suggesting a potential impact on aroma perception. Conversely, CMNP-treated samples contained 236.8 ± 15.2 µg L^−1^ (skimmed) and 165.1 ± 14.2 µg L^−1^ (whole milk), approximately five times higher than the odor threshold, indicating substantial odor attenuation compared with unencapsulated CAR.

Odor retention depends on factors such as molecular weight, volatility, polarity, and the wall material’s structural characteristics [44]. Tópor et al. (2025) [16] previously demonstrated the ability of chia mucilage to mask CAR odor in grapes, where encapsulated samples showed CAR levels below the odor threshold throughout storage. The present study extends this evidence to a dairy matrix, showing for the first time that chia mucilage nanocapsules effectively reduce volatile losses of CAR in milk. However, as no study evaluated the odor threshold of CAR in milk samples, future studies need to be conducted to assure the sensorial implications of the presence of CMNP in this food matrix.

Overall, the results indicate that CAR encapsulated in chia mucilage was gradually released throughout the storage period, in both whole and skimmed milk, under refrigeration (5 °C) and at temperatures favorable to bacterial growth (37 °C). This behavior explains the sustained antimicrobial activity observed and reinforces the technological potential of CMNP for application in dairy products. Furthermore, the choice of wall material directly influences the stability and release of aromatic compounds such as CAR [45].

## 4. Conclusions

CMNP increased the antioxidant activity of CAR in milk and showed greater efficacy than free CAR (CS) in inhibiting *E. coli* growth in both skimmed and whole milk, under both optimal growth conditions (37 °C) and refrigeration (5 °C). The application of CMNP maintained bacterial counts below the detection limit, indicating effective microbial control during storage. In addition, CMNP showed time-dependent changes in measurable CAR levels, in contrast to the more pronounced loss observed for free CAR, supporting its sustained availability in the milk matrix.

Overall, the combined antimicrobial and antioxidant performance of CMNP highlights its potential as a natural strategy to improve the microbiological safety and oxidative stability of dairy products. In addition, the results indicate that, although milk fat content influences CAR availability and antimicrobial performance, it does not appear to be the main factor governing CAR loss during storage.

This study contributes to the current knowledge by providing an integrated evaluation of the behavior of nanoencapsulated carvacrol in dairy systems, including its retention and functional performance under realistic storage conditions. Future studies should assess sensory acceptance and technological feasibility to support potential industrial applications.

## Data Availability

The original contributions presented in this study are included in the article/Supplementary Materials. Further inquiries can be directed to the corresponding author.

## Supplementary Materials

The following supporting information can be downloaded at: https://www.mdpi.com/article/10.3390/foods15071196/s1, Table S1: Bactericidal Inactivation Concentration (BIC) of CS and CMNP against *Escherichia coli*.
