# Supplementary material for: Carvacrol Encapsulation in Chia Mucilage Nanocapsules Enhances Antimicrobial Activity Against Escherichia coli and Preserves Antioxidant Properties in Milk
Source: Foods. 2026 Apr 2;15(7):1196. doi: 10.3390/foods15071196 (PMC13073405; doi:10.3390/foods15071196)
Supplement: Supplementary file 1 [file foods-15-01196-s001.zip › Table S1.pdf]

Table S1. Bactericidal Inactivation Concentration (BIC) of CS and CMNP against *Escherichia coli*.

BIC ($mg\ mL^{-1}$)		
**Strains**	**CS**	**CMNP**
Escherichia coli 25922	0.66 ± 0.00^{c}	0.83 ± 0.00^{b}
Escherichia coli 8739	1.33 ± 0.00^{a}	0.83 ± 0.00^{b}
Escherichia coli DH5 α	1.33 ± 0.00^{a}	0.83 ± 0.00^{b}
E. coli cocktail	1.33 ± 0.00^{a}	0.83 ± 0.00^{b}

CS – Carvacrol in solution. CMNP – Chia mucilage nanocapsules with carvacrol. Different letters in the same row indicate significant differences ($p < 0.05$). Data represent mean ± standard deviation of three experimental replicates.
